# Supplementary material for: Qualification of ELISA and neutralization methodologies to measure SARS-CoV-2 humoral immunity using human clinical samples
Source: J Immunol Methods. 2021 Dec;499:None. doi: 10.1016/j.jim.2021.113160 (PMC8481082; doi:10.1016/j.jim.2021.113160)
Supplement: Supplementary file 1 — Supplementary material [file mmc1.docx]

**Qualification of ELISA and neutralization methodologies to measure SARS-CoV-2 humoral immunity using human clinical samples**

Sasha E. Larsen^1*^, Bryan J. Berube^1,2*^, Tiffany Pecor^1^, Evan Cross^1^, Bryan P. Brown^1^, Brittany Williams^1,3^, Emma Johnson^1^, Pingping Qu^4^, Lauren Carter^5^, Samuel Wrenn^5^, Elizabeth Kepl^5^, Claire Sydeman^5^, Neil P. King^5^, Susan L. Baldwin^1^, Rhea N. Coler^1,3,6#^.

**Supplemental Figures**


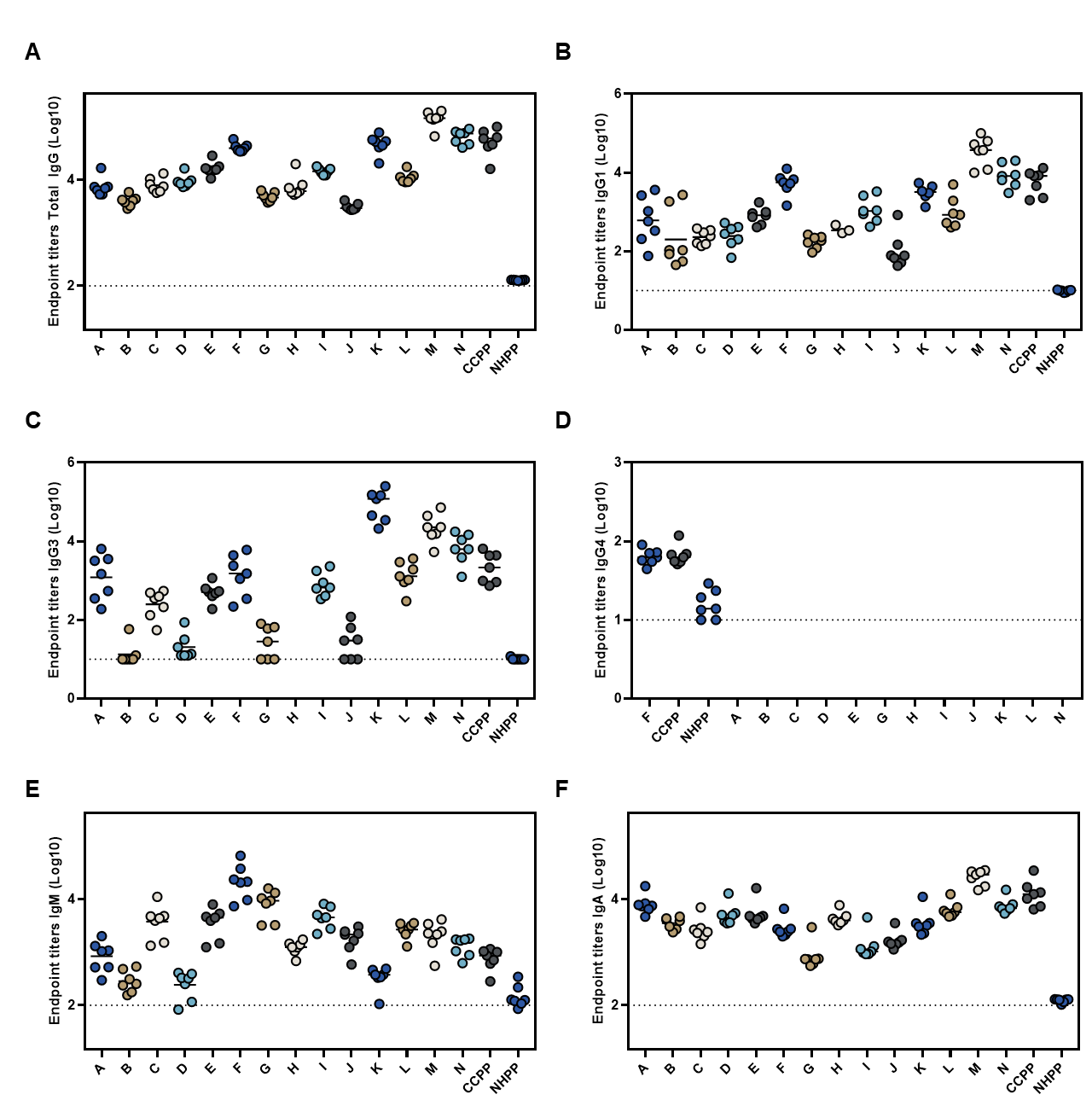


**Supplemental Figure 1: Cumulative EPT across samples from ELISA Precision analysis.** Log 10 cumulative EPT values across individual samples and controls (CCPP, NHPP) for SARS-CoV-2 spike-antigen specific antibody responses by class. A) Total IgG, B) IgG1, C) IgG3, D) IgG4, E) IgM, and F) IgA.


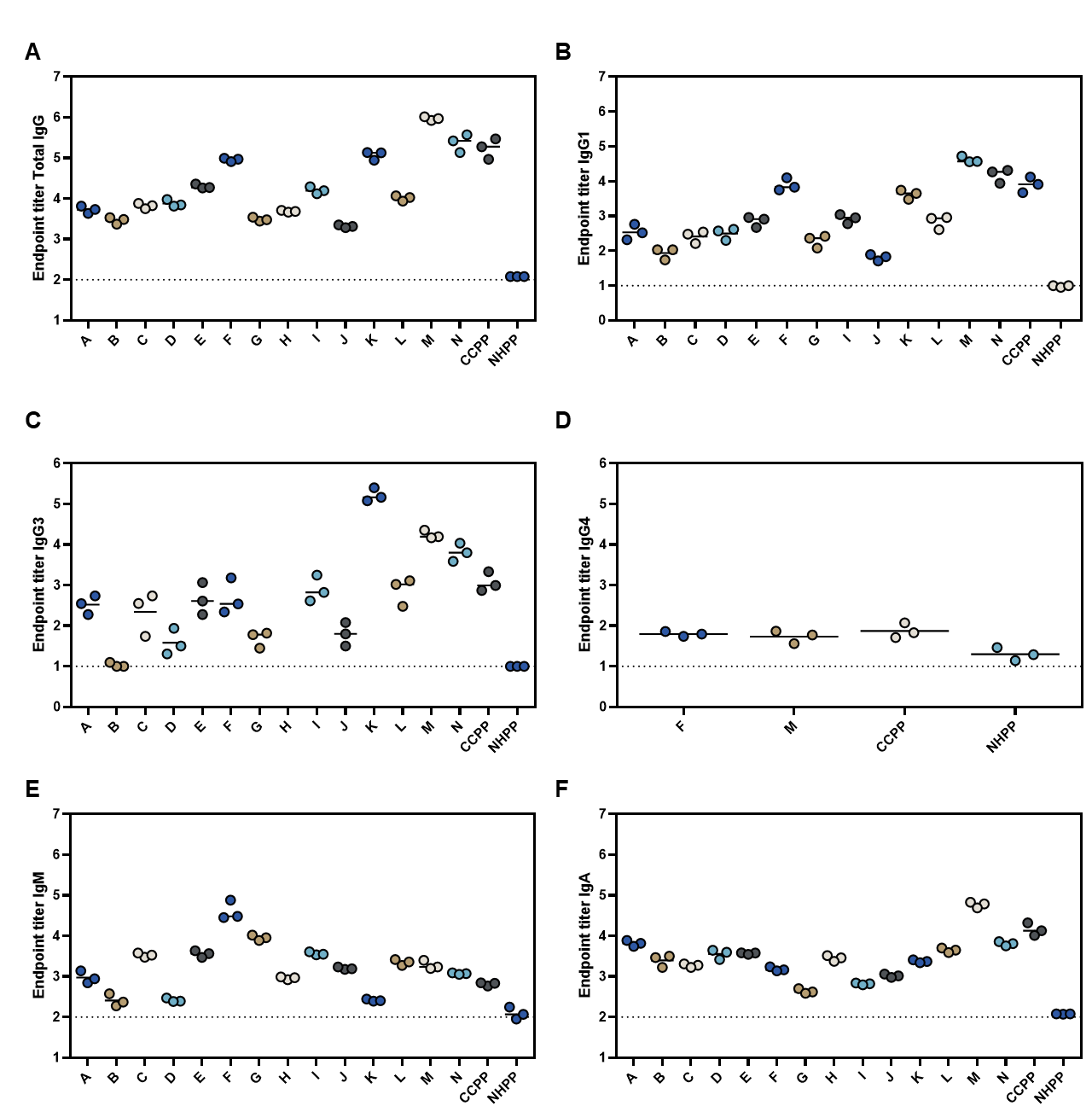


**Supplemental Figure 2: Intraday EPT across samples from ELISA Precision analysis.** Log 10 EPT values from Intraday analysis across individual samples and controls (CCPP, NHPP) for SARS-CoV-2 spike-antigen specific antibody responses by class. A) Total IgG, B) IgG1, C) IgG3, D) IgG4, E) IgM, and F) IgA.


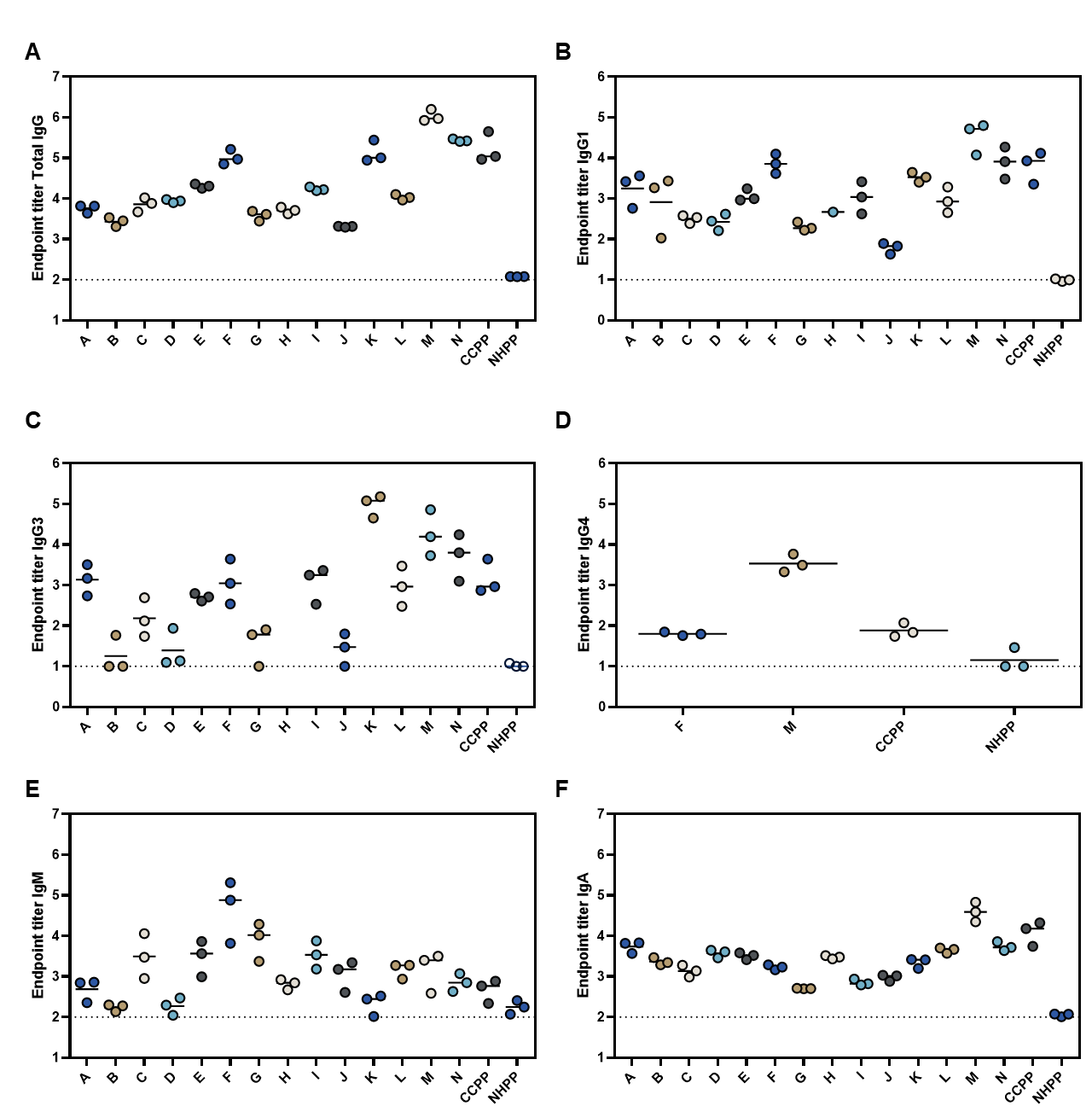


**Supplemental Figure 3: Interday EPT across samples from ELISA Precision analysis.** Log 10 EPT values from Interday analysis across individual samples and controls (CCPP, NHPP) for SARS-CoV-2 spike-antigen specific antibody responses by class. A) Total IgG, B) IgG1, C) IgG3, D) IgG4, E) IgM, and F) IgA.


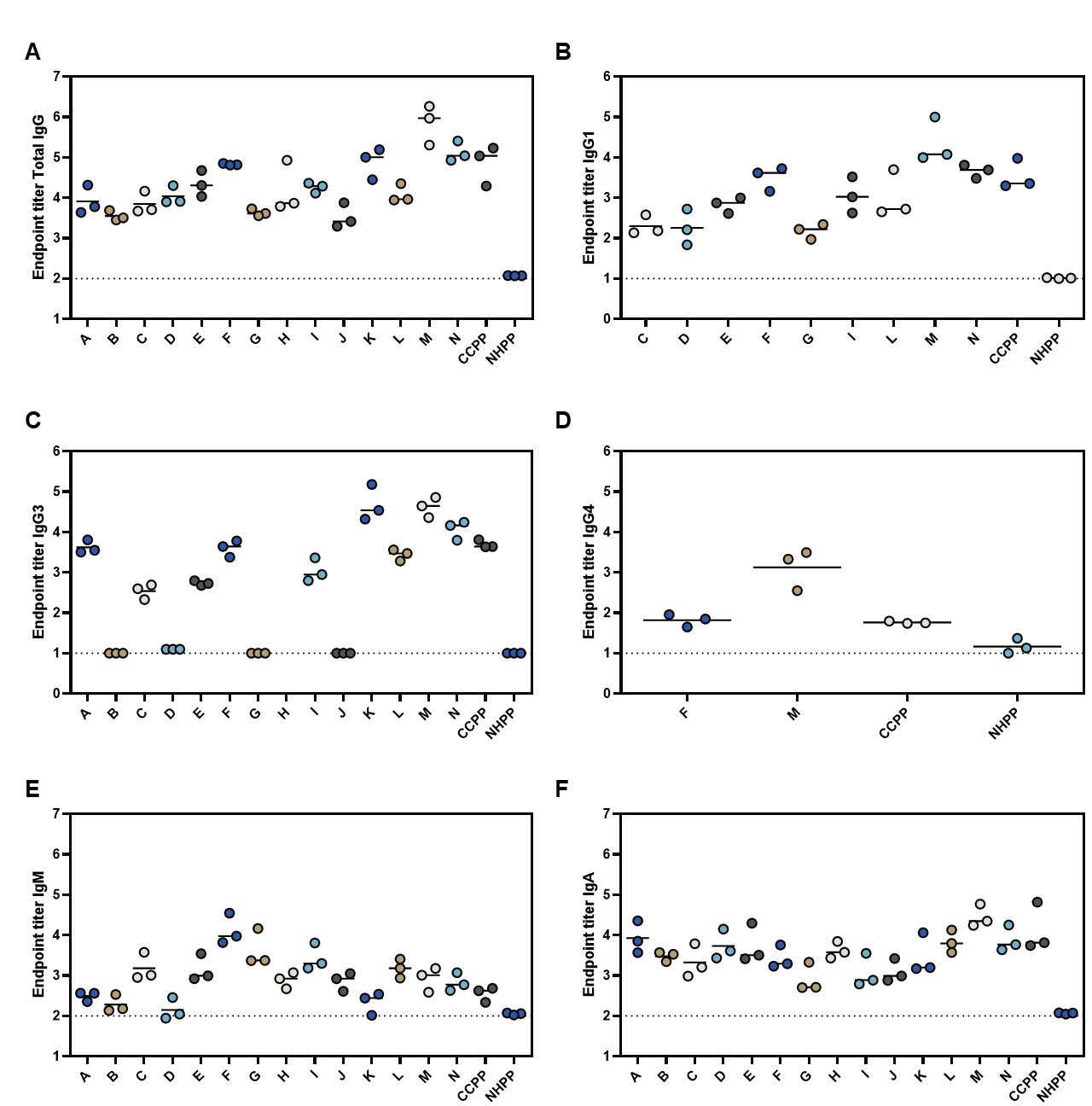


**Supplemental Figure 4: Interoperator EPT across samples from ELISA Precision analysis.** Log 10 EPT values from Interoperator analysis across individual samples and controls (CCPP, NHPP) for SARS-CoV-2 spike-antigen specific antibody responses by class. A) Total IgG, B) IgG1, C) IgG3, D) IgG4, E) IgM, and F) IgA.


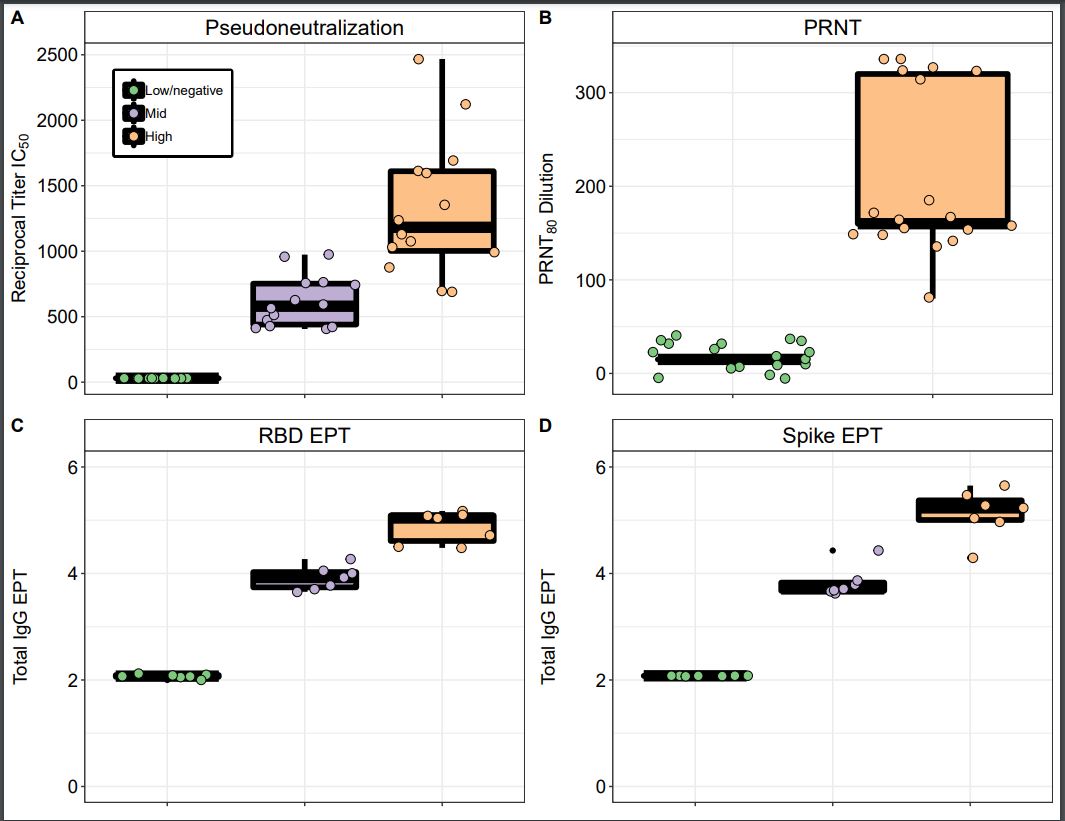


**Supplemental Figure 5:** **High concordance of responses between EPT, Pseudovirus and PRNT assays.** High (orange), mild (purple) and low/negative (green) samples were evaluated across A) Pseudovirus neutralization, B) live virus PRNT, C) RBD EPT ELISA and D) Spike EPT ELISA. Data shown represent individual samples run across each assay.
